# BEREAVEMENT IN A FAMILY CONTEXT

**DOI:** 10.1093/geroni/igz038.2247

**Published:** 2019-11-08

**Authors:** Jeffrey E Stokes, Kyungmin Kim, Deborah Carr

**Affiliations:** 1 University of Massachusetts Boston, Boston, Massachusetts, United States; 2 Boston University, Boston, Massachusetts, United States

## Abstract

Bereavement is an impactful, often difficult experience for individuals throughout the life course. Moreover, bereavement experiences inherently involve wider family networks: The death of a spouse is often also the death of a parent, grandparent, or sibling, as well. The present symposium investigates a variety of different family loss experiences that individuals are exposed to in adulthood and older age, and situates such bereavement in a larger family context. Stahl explores how daily routines and sleep patterns can be altered by spousal bereavement, and assesses an intervention designed to improve widowed older adults’ behaviors and, in turn, reduce their depressive symptomology. Kim and colleagues analyze the death of a parent in adulthood, examining the extent to which pre-loss relationship quality and relationship importance may predict post-loss symptoms of grief. Stokes and colleagues extend this intergenerational perspective, examining the death of a grandparent in adulthood, and whether adult grandchildren’s relationships with their middle-generation parents – bereaved adult children themselves – impact their experiences of grief after loss. Focus is also paid to the influence of gender across all three generations. Lastly, Donnelly explores the cumulative consequences of experiencing multiple family deaths throughout the life course for adults’ health trajectories. Together, these papers expand the scope of bereavement research to incorporate spousal, multigenerational, and cumulative loss experiences and their repercussions for midlife and older adults. As discussant, Carr will assess the contributions of these papers to theory and the literature, and highlight potential directions for future research concerning aging, families, and bereavement.

